# The Book World of Medicine and Science

**Published:** 1896-12-12

**Authors:** 


### The Book World of Medicine and Science.

Manual of Diseases of the Ear. By Thomas Barr, M.D. Second edition. (Glasgow: James Maclehose and Sons. 1896.)

Many alterations are to be noticed on comparing this edition with the first, and they are without exception such as to make it a far more readable book for students, and for practitioners one more easy to refer to for particular information in those cases which come under their care. Head lines, and separation into smaller paragraphs, as well as the inclusion of a number of new illustrations, are the chief features which distinguish it from the first edition. The principles of treatment are such as are generally taught in the metropolitan schools, although there are naturally points on which all authorities would not be agreed. For instance, in regard to the question of perforation of the tympanic membrane, in London many aural surgeons have no hesitation in incising merely on the suspicion of fluid, and in children and infants on very meagre diagnostic evidence. Mr. Barr is most careful in his teaching on this point. Although advising incision when severe pain exists in the ear, attended by recent and marked loss of hearing, in the absence of confirmatory signs and symptoms, he considers that puncture is contra-indicated when the membrane is simply acutely inflamed in the absence of localised yellowish bulging.

With regard to that difficult question of tinnitus, the student will seek in vain for a definite explanation of many of its diverse forms. For instance, in anæmia, Mr. Barr remarks that it is apt to occur through the anæmic state of the labyrinth; and, again, that the "jugular vein under the floor of the tympanum is often the source of the sound," in fact, that a "fluid vein" exists in the situation when the vascular contents are decreased in anæmic conditions. In a small type paragraph in page 363 an interesting sidelight is thrown on the question of nystagmus, which has received at various times and from various sources many different explanations. In miners it is of comparatively frequent occurrence, and owing to the high atmospheric pressure at which they work, it might possibly be produced from this cause, the pressure in the sub-arachnoid cavity being increased through aqueducts of the vestibule and cochlea, secondarily causing irritation of the abducens at the base of the brain. There can be no question but that this new edition will be well received by practitioners and students, as it deals with every question from the practical side, and leaves out as far as possible unfruitful controversial discussion.

